# Effectiveness of nifedipine, labetalol, and hydralazine as emergency antihypertension in severe preeclampsia: a randomized control trial

**DOI:** 10.12688/f1000research.125944.2

**Published:** 2023-04-27

**Authors:** Donel S, Dhini Aiyulie Novri, Yulis Hamidy, Maya Savira

**Affiliations:** 1 Department of Obstetrics and Gynaecology, Faculty of Medicine, Universitas Riau, Pekanbaru, 28293, Indonesia; 2 Department of Pharmacology, Faculty of Medicine, Universitas Riau, Pekanbaru, 28293, Indonesia; 3 Department of Microbiology, Faculty of Medicine, Universitas Riau, Pekanbaru, 28293, Indonesia

**Keywords:** Hypertension; gestation; systolic blood pressure; diastolic blood pressures; mean arterial pressure; preeclampsia

## Abstract

**Background**: Preeclampsia is a highly prevalent disease among pregnant women. In the event of hypertensive emergency, nifedipine, labetalol, and hydralazine are assigned as first-line therapies in preeclampsia. Further studies are needed to compare the effectiveness of these drugs to find the most cost-effective drug with minimal side effects. This study aimed to compare the effectiveness of these drugs in lowering blood pressure during hypertensive emergencies in severe preeclampsia.

**Methods**: 60 pregnant women with severe preeclampsia were recruited in this multiple centre double-blind randomized clinical trial from May 2021 to April 2022 in Indonesia. The patients were divided equally into three groups and treated with three doses of nifedipine, labetalol, and hydralazine, respectively within one hour with 20 minutes interval. The effectiveness was measured based on systolic and diastolic blood pressures, and mean arterial pressure (MAP). The observation was carried out until five hours post-third dose administration.

**Results**: The blood pressure was reduced significantly after the administration of the first to the third dose of each antihypertensive (p<0.05). A single dose administration, four, one, and three patients had 20% MAP reduction in nifedipine, labetalol, and hydralazine group. Three, seven, and one patient had a failure of reaching 20% MAP reduction even after receiving the third dose. The effectiveness of the drugs to achieve 20% reduction of MAP could be ranked as follows: nifedipine>labetalol>hydralazine (57.49%, 42.13%, and 40.87%, respectively) for single dose and hydralazine>nifedipine>labetalol (111.3%, 85.12%, and 90.04%, respectively) for triple dose.

**Conclusions**: Nifedipine is the most effective drug to reduce the blood pressure when single dose administration is used, but requires more doses to further reduce the blood pressure. Hydralazine is the most effective when the drug administration is maxed up to three doses within 60 minutes with 20 minutes interval.

**Thai Clinical Trials Registry (TCTR):** TCTR20221014007 (14/10/2022)

## Introduction

Preeclampsia and eclampsia are major problems in obstetric cares. Preeclampsia is one of the main causes of maternal morbidity and mortality in addition to infection and bleeding with the prevalence rate between 5–8% among pregnant women. ^1^ The main cause of preeclampsia is unknown and the disease is therefore also called the disease of theory. As a result, the guidelines of preeclampsia management are updated and changed from time to time. ^2^ Research continues to be updated to find the best formulation for diagnosis and management with the main aim to provide the management with the most optimal outcome for the mother and foetus. Termination of pregnancy is still considered the gold standard in severe preeclampsia, but this becomes a problem when the pregnancy is still premature as it can increase the infant mortality rate. ^1^

High blood pressure is the main indicator in the diagnosis of preeclampsia. Treatment of severe preeclampsia requires anticonvulsant and antihypertensive drugs. The purpose of administering antihypertensive drugs is to prevent cerebral bleeding. The American Congress of Obstetricians and Gynaecologists (ACOG) states that pregnant women with systolic blood pressure 160 mmHg and/or diastolic 110 mmHg that persist for 15 minutes should be considered an emergency condition and receive antihypertensives immediately, no later than 30 minutes. ^2^ To date, ACOG has offered nifedipine, labetalol, or hydralazine as the antihypertensive emergency in preeclampsia. ^2^ In June 2020, ACOG issued a statement that women with gestational hypertension presenting with high blood pressure (severe range) should be treated as same as severe preeclampsia. ^1^

An indicator of a decrease in blood pressure in patients with severe preeclampsia is the mean arterial pressure (MAP). MAP is obtained by the formula of twice the diastolic pressure plus one systolic pressure, then divided by three. ^3^ The target for MAP reduction in patients with severe preeclampsia is around 20–25%. ^3^ This aims to maintain adequate uteroplacental circulation for the foetus. If blood pressure is reduced too low, there will be a sharp decrease in placental perfusion resulting in foetal distress, while the main goal of the management of preeclampsia is not only centred on the mother, but also on the outcome of the baby.

Recommendations for antihypertensive drugs in preeclampsia are changing rapidly. Multiple antihypertensive drugs have been listed as drugs of choice for the treatment of severe preeclampsia but then excluded due to inconsistent treatment outcomes. Intravenous labetalol and intravenous hydralazine have long been considered as first-line therapy in hypertensive emergencies, severe hypertension in pregnant women, and in the postpartum period. ^4^ Although there is little information regarding the use of calcium channel blockers, several studies have shown that oral nifedipine can also be considered as first-line therapy, especially when intravenous access is difficult. ^2^ However, further studies are needed to compare the effectiveness of these three drugs with the aim of finding cost-effective drugs with minimal side effects, and right on target.

Previous studies on the effectiveness of these three drugs showed mixed results. ^4^^,^^5^ Hydralazine and labetalol are considered to have a balanced effectiveness and side effect to achieve target blood pressure (systolic blood pressure 140–150 mmHg and diastolic blood pressure 90–100 mmHg) in preeclampsia. ^4^^,^^6^ However, labetalol lowered blood pressure more rapidly than hydralazine. ^4^ Another study comparing nifedipine and labetalol showed that these two drugs had the same effectiveness in achieving target blood pressures (systolic blood pressure 120–150 mmHg and diastolic blood pressure 70−100 mmHg) within 6 h with no adverse outcomes; 84% women vs 77% women with p=0.05. ^5^ However, single-use nifedipine was more effective than labetalol. ^5^

In our preliminary study, the emergency antihypertensive drugs in severe preeclampsia that are often used in the Arifin Achmad Hospital, Riau Province of Indonesia and the affiliated hospitals of Faculty of Medicine, Universitas Riau, are nifedipine and methyldopa. There has never been a standardized study or observation on the safety and effectiveness of antihypertensive drugs in Indonesian population. Therefore, this study was conducted to compare the effectiveness of oral nifedipine, and intravenous labetalol and intravenous hydralazine as antihypertensives in severe preeclampsia.

## Methods

### Study setting

This study was a double-blind, randomized clinical trial to compare the effectiveness of oral nifedipine, intravenous labetalol, and intravenous hydralazine as antihypertensive emergency in severe preeclampsia. The multicentre study was conducted at four hospitals at Riau Province of Indonesia, Arifin Achmad Hospital, Tengku Rafian Siak Sri Indrapura Hospital, Dumai Hospital, and Bengkalis Hospital, from May 2021 to April 2022. These hospitals were chosen because they are affiliated with Faculty of Medicine, Universitas Riau.

### Ethical considerations and registration

The protocol of the study was approved on 28 May 2021 by the Medical and Health Research Ethics Unit, Faculty of Medicine, Universitas Riau with the number B/045/UN19.5.1.1.8/UEPKK/2021. This clinical trial has been registered at Thai Clinical Trials Registry (TCTR) with identification number is TCTR20221014007 (approved on 14 October 2022). This clinical trial was registered retrospectively in TCTR since the protocol of the study has been registered locally and this following the Indonesia law. All patients gave written informed consent before the enrolment. This study is reported in line with the CONSORT guidelines. ^20^

### Patient recruitment and criteria

The severe preeclampsia patients were recruited consecutively from populations that met the inclusion criteria. The minimal sample size of the study was 13 patients for each arm. This number based on calculation based on formula:

$$n1=n2-n3={\left[\frac{\left(\mathrm{Z\alpha}+\mathrm{Z\beta}\right)S}{\left(x1-x2\right)}\right]}^{2}\;\left(1-\rho \right)$$

where α is the significance level to be measured (0.05), β is the study power (80%) and *x*1- *x*2 is the difference of the score between groups that considered significant (10) and S is standard deviation e between groups.

However, a total of 20 severe preeclampsia patients were recruited for each group to prevent underpower due to drop-out and therefore there were 60 patients included in this study. The severe preeclampsia was defined as pregnant women with systolic blood pressure ≥160 mmHg or diastolic ≥110 mmHg that persisted for 15 minutes. Inclusion criteria were: (a) severe preeclampsia patient with 28–34-week gestation; (b) had live foetus; (c) upper arm circumference 23.5–33 cm; and (d) haemoglobin level at least 10.5 g/dL. All severe preeclampsia patients with decreased consciousness; had complications such as eclampsia, HELLP syndrome, kidney failure, or acute pulmonary oedema; in labour; received antihypertensive therapy in the last 12 hours; had an allergy to the trial drugs; and had asthma and heart disease were excluded. In this study the drop-out criteria were: the mother experienced allergy symptoms, had labour, or emergency symptoms such as placental abruption and foetal distress during the study.

### Blinding and study procedure

All severe preeclampsia patients who came to the hospitals were subjected to anamnesis, physical and laboratories examination and then selected based on predetermined inclusion criteria. Blood pressure measurements were carried out using a digital sphygmomanometer with the Omron 7600T brand. The patient was asked to sit and rest for 10 minutes, the measurement was conducted twice on the arm with a distance period of 15 minutes, and the data taken was the second measurement. If the patients met the requirements, they were asked to participate in the study and all patients have to sign the written informed consent before the enrolment. The assigned group was computerized randomized using Randomizer software. Simple randomization was employed. In brief, 60 kits were prepared by the authors and were labelled 1 to 60 and each of kit containing three tables and three ampoules with the same sizes and colours. Kits no 1–20, 21–40 and 41–60 contained nifedipine, labetalol and hydralazine, respectively. For example, for one nifedipine kit, it contained 3 nifedipine tablets and 3 ampoules of placebo liquid. An author created the random sequence list consisting who will receive the kit no 1 until 60 based on random sequences provided by Randomizer software. For example, if the list from the software run was 41, 23, 4 and soon, the patient who arrived first, second and third to the hospital would be given kit no 41, 23 and 4, respectively. Each drug was administered 3 doses within 60 minutes (i.e., 20 minutes interval). Each dose of the drug contained: oral nifedipine (20 mg), intravenous labetalol (20 mg in 10 ml of sodium chloride 0.9%) and intravenous hydralazine (10 mg in 10 ml of sodium chloride 0.9%). Since the study consisted multiple healthcare centers, patient enrolment was conducted in one hospital at one time based on allocated time. Therefore, there were no separation of the sequence list between centres.

The enrolment of the patient was conducted by the doctors in each centre. The doctors have been informed about the study and have been trained. The doctors and patients did not know the drugs contained in the kit. The doctors were instructed to administrate one pair of drugs (i.e., one tablet and one ampoule injection; this contained one drug and one placebo) for each time. The blood pressure was assessed 20 minutes after each administration. If the blood pressure did not meet the MAP target, the second pair of drugs (one tablet and one ampoule injection) was given and the blood pressure was reassessed in 20 minutes until the third pair of the drugs. The blood pressure was re-assessed 60 min post-first dose, 2, 4 and 6 h post-first dose. Therefore, the blood pressure was measured seven times as follows: (1) minute 0 (pre-treatment); (2) 20 min post-first dose; (3) 20 min post-second dose (i.e., 40 min post-first dose); (4) 20 min post-third dose (i.e., 60 min post-first dose); (5) 2 h post-first dose; (6) 4 h post-first dose; and (7) 6 h post-first dose.

The technique of administering magnesium sulphate and dexamethasone was uniform in all patients. If the MAP was nor reached after the third dose, the patient was treated with other drugs and excluded from the final analysis.

### End points

The end points of this study were: (a) systolic blood pressure; (b) diastolic blood pressure; (c) MAP; and (d) side effects.

### Statistical analysis

Characteristics of patients from each group were tested for normality. The normality of data was measured using the Kolmogorov-Smirnov test. The data that had normal distribution were analysed using one-way ANOVA and followed by post hoc analyses. The data that did not distribute normally were analysed using Kruskal-Wallis and Friedmann followed by post hoc analyses. Comparison of the effectiveness of the three drugs was tested using N-Gain analysis. To determine how far the differences exist, Cohen's d effect size analysis was used. Further analysis with Kaplan-Meier survival was conducted. Statistical analysis was performed using SPSS software version 22 (Statistical Package for Social Sciences, Chicago, IL, USA).

## Results

**Figure 1.**
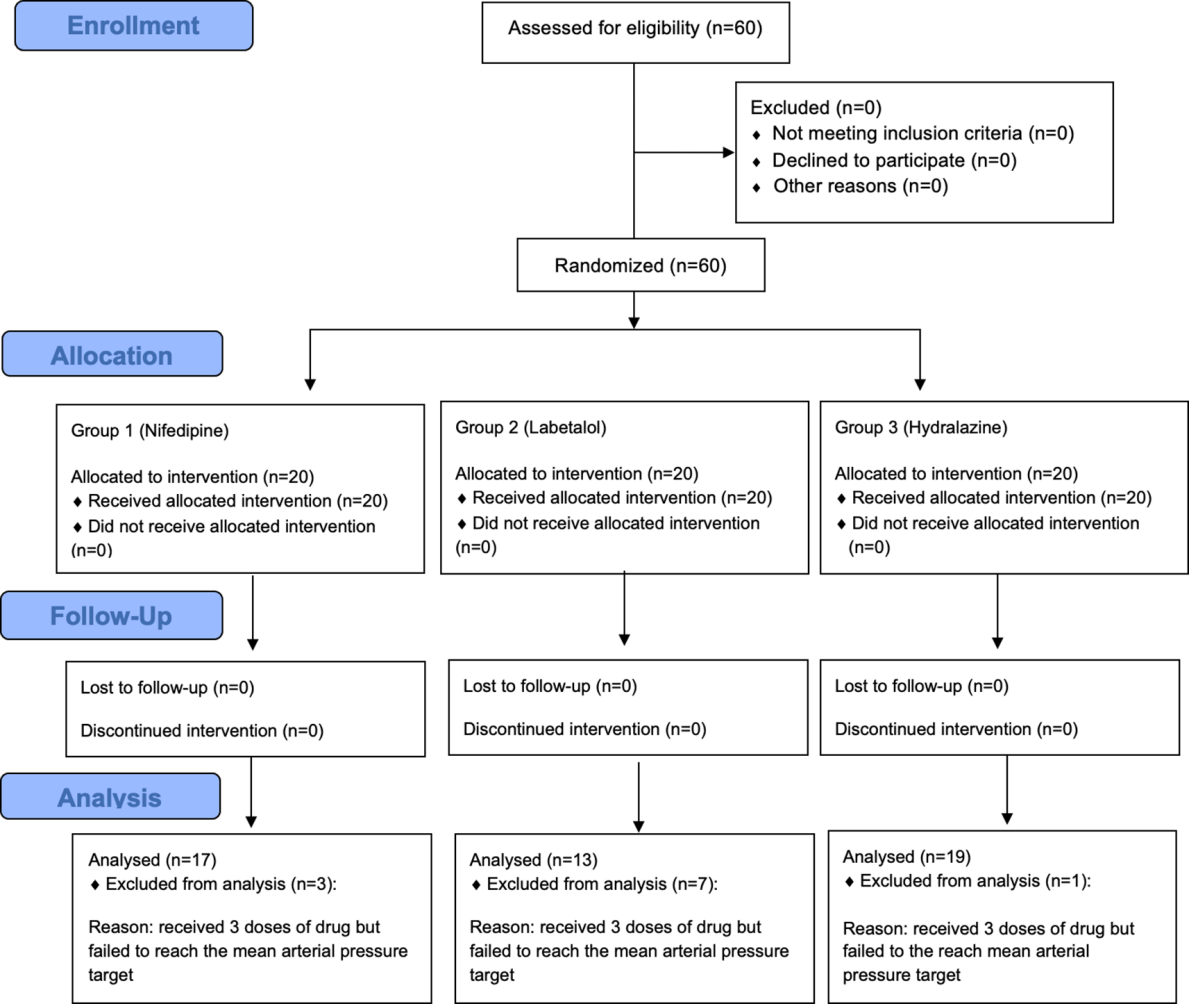
Flowchart of patients’ enrolment, allocation, follow-up and analysis.

The study was conducted on 60 patients with severe preeclampsia and they were divided into 3 groups: 20 patients in the nifedipine group, 20 patients in the labetalol group, and 20 patients in the hydralazine group. ^19^ Patients were recruited from the population that met the inclusion criteria and were willing to participate in the study by signing an informed consent. Patients were then monitored closely and periodically according to the study protocol. The number of patients for each step of enrolment, allocation, follow-up and analysis are presented in Figure 1. Out of 60 patients, 11 patients did not achieve the MAP target after three doses of the tested drugs and therefore were excluded for effectiveness analysis of nifedipine, labetalol, and hydralazine to reduce the blood pressure.

### Patients’ characteristics

We recruited 60 severe preeclampsia patients and were divided randomly to receive nifedipine, labetalol and hydralazine. The characteristics of the patients including age, gravida, gestational age (gestation), upper arm circumference (UAC), systolic, diastolic, MAP, pulse, and foetal heart rate of each group are presented in Table 1. Our data indicated that there is uniformity of characteristics between the three groups.

**Table 1.** Characteristics of the patients of three study groups pre-treatment.

Characteristic	Nifedipine *n*=20	Labetalol *n*=20	Hydralazine *n*=20	p-value ^a^
Age (year)	30.7±5.8	30.3±5.2	30.9±4.2	0.148
Gravida	1.6±0.9	1.5±0.7	1.5±0.6	0.142
Gestation (week)	31.2±3.0	31.7±1.8	31.5±3.6	0.227
Upper arm circumference (mm)	30.7±3.3	28.6±2.3	28.9±2.4	0.588
Systolic blood pressure (mmHg)	170.7±11.6	177.4±16.0	174.2±13.7	0.088
Diastolic blood pressure (mmHg)	112.6±19.4	105.7±15.8	107.7±16.0	0.645
Mean arterial pressure (mmHg)	132.0±14.5	130.0±12.0	130.0±9.6	0.094
Pulse (min ^-1^)	83.4±1.8	83.2±1.2	85.5±1.4	0.260
Foetal heart rate (min ^-1^)	146.4±7	144.3±6.9	144.6±5.4	0.346

^a^ Analysis was conducted using ANOVA.

### Required dose for nifedipine, labetalol, and hydralazine to achieve MAP target

**Figure 2.**
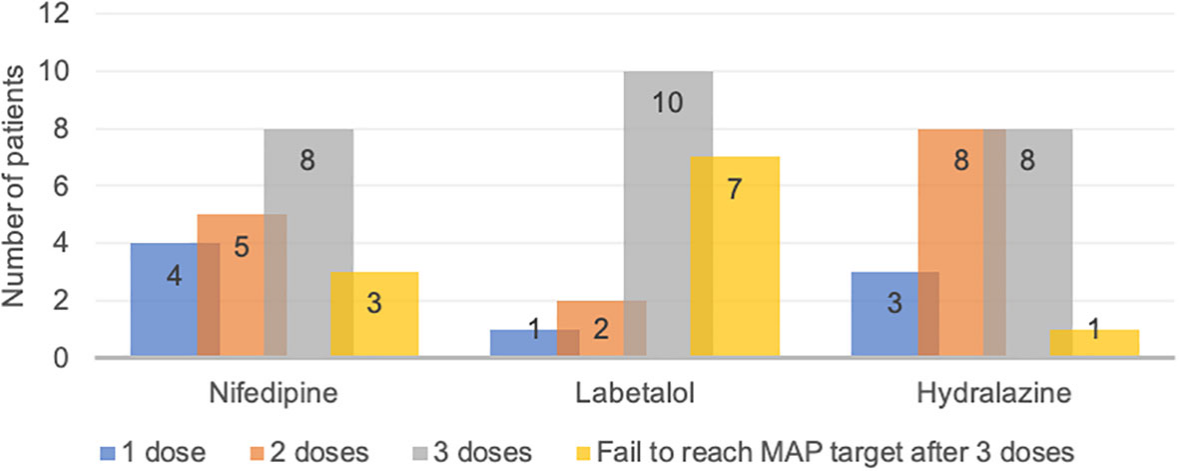
Dose required to reach target mean arterial pressure (MAP) in patients with severe preeclampsia treated with nifedipine, labetalol, and hydralazine.

Data regarding the dose required for nifedipine, labetalol, and hydralazine to reach the target MAP (20% reduction) are presented in Figure 2. The study found that 4, 1 and 3 patients of nifedipine, labetalol, and hydralazine group achieved the MAP target with only one dose, respectively. There were 3, 7 and 1 patients were unable to achieve the target MAP after three doses of nifedipine, labetalol, and hydralazine, respectively and these patients were excluded from further analyses.

### Effectiveness of nifedipine, labetalol, and hydralazine to reduce the blood pressure

We assessed the effectiveness of nifedipine, labetalol, and hydralazine to reduce the systolic and diastolic blood pressure where the data were treated as numerically to provided more detailed blood pressure reduction.

***Nifedipine***

The effectivity of nifedipine to reduce the systolic, diastolic and MAP is presented in Table 2. Our data indicated that nifedipine decreased the systolic pressure the most at 20 min post-first dose (difference from the first measurement to the second measurement) as much as 13.41 mmHg with a p<0.001. Meanwhile for second dose (second third measurement), and the next dose no longer showed a significant difference. Nifedipine also decreased diastolic pressure the most and significantly at 20 min post-first dose as much as 16.71 mmHg with a p<0.001. Nifedipine decreased the MAP 15.61 mmHg, 6.37 mmHg and 2.90 mmHg 20 min post the first, second and the third dose of nifedipine. The MAP reduction on 20 min post the first dose was significant statistically with p<0.001.

**Table 2.** Effect of nifedipine to reduce the blood pressure over time in severe preeclampsia patient.

Blood pressure	Time comparation	Mean difference (I-J) ^a^	95% CI for difference ^b^		p-value ^b^
			Lower bound	Upper bound	
Systolic	Pre – 20 min	13.412	7.019	19.805	<0.001 ^*^
	20 min – 40 min	4.412	-4.464	13.288	1.000
	40 min – 60 min	3.765	-4.857	12.387	1.000
	60 min – 2 h	4.529	-14.701	23.760	1.000
	2 h – 4 h	-4.176	-10.050	1.697	0.438
	4 h – 6 h	-7.765	-16.654	1.124	0.131
	6 h – Pre	-14.176	-23.586	-4.767	0.001 ^*^
Diastolic	Pre – 20 min	16.706	9.634	23.778	<0.001 ^*^
	20 min – 40 min	7.353	-1.143	15.849	0.139
	40 min – 60 min	2.471	-5.993	10.934	1.000
	60 min – 2 h	2.118	-8.781	13.016	1.000
	2 h – 4 h	-3.706	-10.410	2.999	1.000
	4 h – 6 h	-7.118	-12.873	-1.362	0.008 ^*^
	6 h – Pre	-17.824	-31.400	-4.247	0.005 ^*^
MAP	Pre – 20 min	15.608	10.391	20.825	<0.001 ^*^
	20 min – 40 min	6.373	-0.925	13.670	0.131
	40 min– 60 min	2.902	-5.090	10.894	1.000
	60 min – 2 h	2.922	-10.434	16.277	1.000
	2 h – 4 h	-3.863	-9.050	1.324	0.343
	4 h – 6 h	-7.333	-12.962	-1.705	0.005 ^*^
	6 h – Pre	-16.608	-26.980	-6.236	0.001 ^*^

^a^ Based on estimated marginal means.

^b^ Adjustment for multiple comparisons: Bonferroni.

* The mean difference is significant at p<0.01.

**Figure 3.**
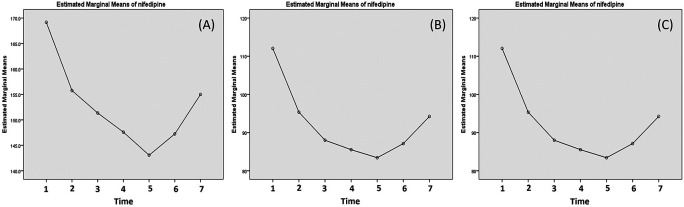
Changes of systolic (A), diastolic (B) and mean arterial pressure (MAP) post nifedipine administration. The blood pressure is measured: (1) minute 0 (pre-treatment); (2) 20 min post-first dose; (3) 20 min post-second dose; (4) 20 min post-third dose; (5) 1 h post-third dose; (6) 3 h post-third dose; and (7) 5 h post-third dose.

The changes of the mean systolic, diastolic and MAP are presented in Figure 3. The data suggested there was a sharp decrease of systolic and diastolic after the first dose of nifedipine and the blood pressure kept decrease until 20 min after the third dose (after 2 hours of first dose) but it started to bounce again afterward ( Figure 3).

***Labetalol***

**Figure 4.**
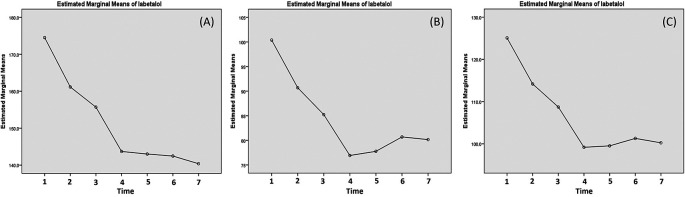
Changes of systolic (A), diastolic (B) and mean arterial pressure (MAP) post labetalol administration. The blood pressure is measured: (1) minute 0 (pre-treatment); (2) 20 min post-first dose; (3) 20 min post-second dose; (4) 20 min post-third dose; (5) 1 h post-third dose; (6) 3 h post-third dose; and (7) 5 h post-third dose.

Labetalol decreased systolic blood pressure the most and significantly at 20 min post the first and the third dose (13.38 and 12.00 mmHg) ( Table 3). The systolic blood pressure continued to decrease until 5 hours post the third dose ( Figure 4). The pattern of the of the reduction was similar with diastolic where the reduction mainly occurred after 20 min post the first and third dose (with reduction about 9.69 and 8.30 mmHg, respectively) ( Table 3). In contrast, the diastolic blood pressure increased after the 20 min post the third dose ( Figure 4). The changes of the MAP were similar with diastolic and the increase of diastolic and MAP were very small after the third dose ( Figure 4).

**Table 3.** Effect of labetalol to reduce the blood pressure over time in severe preeclampsia patient.

Blood pressure	Time comparation	Mean difference (I-J) ^a^	95% CI for difference ^b^		p-value ^b^
			Lower bound	Upper bound	
Systolic	Pre – 20 min	13.385	2.538	24.232	0.010 ^**^
	20 min – 40 min	5.462	-8.580	19.503	1.000
	40 min – 60 min	12.000	1.158	22.842	0.024 ^*^
	60 min – 2 h	0.692	-6.271	7.656	1.000
	2 h – 4 h	0.538	-5.423	6.500	1.000
	4 h – 6 h	2.077	-2.097	6.251	1.000
	6 h – Pre	-34.154	-46.904	-21.404	<0.001 ^**^
Diastolic	Pre – 20 min	9.692	3.625	15.759	0.001 ^**^
	20 min – 40 min	5.462	-8.012	18.935	1.000
	40 min – 60 min	8.308	2.609	14.006	0.002 ^**^
	60 min – 2 h	-0.846	-7.149	5.456	1.000
	2 h – 4 h	-2.923	-8.335	2.489	1.000
	4 h – 6 h	0.538	-4.307	5.384	1.000
	6 h – Pre	-20.231	-37.391	-3.070	0.015 ^*^
MAP	Pre – 20 min	10.921	4.931	16.910	<0.001 ^**^
	20 min – 40 min	5.464	-5.311	16.240	1.000
	40 min – 60 min	9.538	2.442	16.635	0.005 ^**^
	60 min – 2 h	-0.333	-6.081	5.414	1.000
	2 h – 4 h	-1.769	-5.590	2.052	1.000
	4 h – 6 h	1.051	-2.347	4.450	1.000
	6 h – Pre	-24.872	-36.993	-12.751	<0.001 ^**^

^a^ Based on estimated marginal means.

^b^ Adjustment for multiple comparisons: Bonferroni.

* The mean difference is significant at p<0.05.

** The mean difference is significant at p<0.01.

***Hydralazine***

**Figure 5.**
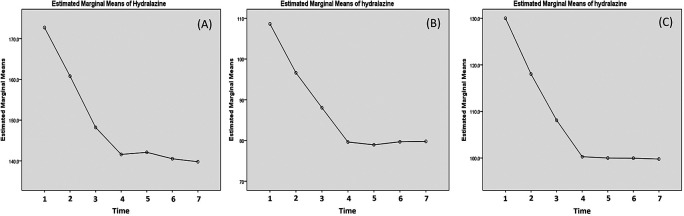
Changes of systolic (A), diastolic (B) and mean arterial pressure (MAP) post hydralazine administration. The blood pressure is measured: (1) minute 0 (pre-treatment); (2) 20 min post-first dose; (3) 20 min post-second dose; (4) 20 min post-third dose; (5) 1 h post-third dose; (6) 3 h post-third dose; and (7) 5 h post-third dose.

Effectivity of hydralazine in lowering the blood pressure has been presented in Table 4. Due to abnormal distribution of the systolic data, Friedman's test was carried out instead. The test yielded p-value of less than 0.001. As for the diastolic blood pressure and MAP, significant decreases was obtained until 20 minutes post-third dose. This can be observed in the curve presented in Figure 5, where sharp decreases of systolic pressure were observed up to 20 minutes post-third administration. However, in the case of systolic blood pressure, a mild rebound occurred at 1 hour post-third administration ( Figure 5).

**Table 4.** Effect of hydralazine to reduce the blood pressure over time in severe preeclampsia patients.

Blood pressure	Time comparation	Mean rank or mean difference (I-J) ^a^	95% CI for difference ^b^		p-value ^b^
			Lower bound	Upper bound	
Systolic	Pre – 20 min	6.95			<0.001 ^c^ ^,^ **
	20 min – 40 min	5.71			
	40 min 3 – 60 min	3.85			
	60 min – 2 h	2.84			
	2 h – 4 h	3.13			
	4 h – 6 h	2.92			
	6 h – Pre	6.95			
Diastolic	Pre – 20 min	12.000	3.928	20.072	0.001 **
	20 min – 40 min	8.579	1.175	15.983	0.014 *
	40 min 3 – 60 min	8.421	1.412	15.430	0.010 *
	60 min – 2 h	0.684	-4.275	5.643	1.000
	2 h – 4 h	-0.737	-3.965	2.491	1.000
	4 h – 6 h	-0.105	-4.567	4.357	1.000
	6 h – Pre	-28.842	-37.887	-19.798	<0.001 **
MAP	Pre – 20 min	11.982	4.784	19.180	<0.001 **
	20 min – 40 min	9.912	3.542	16.283	0.001 **
	40 min 3 – 60 min	7.825	2.416	13.233	0.002 **
	60 min – 2 h	0.281	-3.201	3.762	1.000
	2 h – 4 h	0.035	-2.152	2.222	1.000
	4 h – 6 h	0.175	-2.685	3.036	1.000
	6 h – Pre	-30.211	-34.932	-25.489	<0.001 **

^a^ Based on estimated marginal means.

^b^ Adjustment for multiple comparisons: Bonferroni.

^c^ Analysed using Friedmann test.

* The mean difference is significant at p<0.05.

** The mean difference is significant at p<0.01.

### Effectiveness comparisons of nifedipine, labetalol, and hydralazine after single and triple dose administration

**Figure 6.**
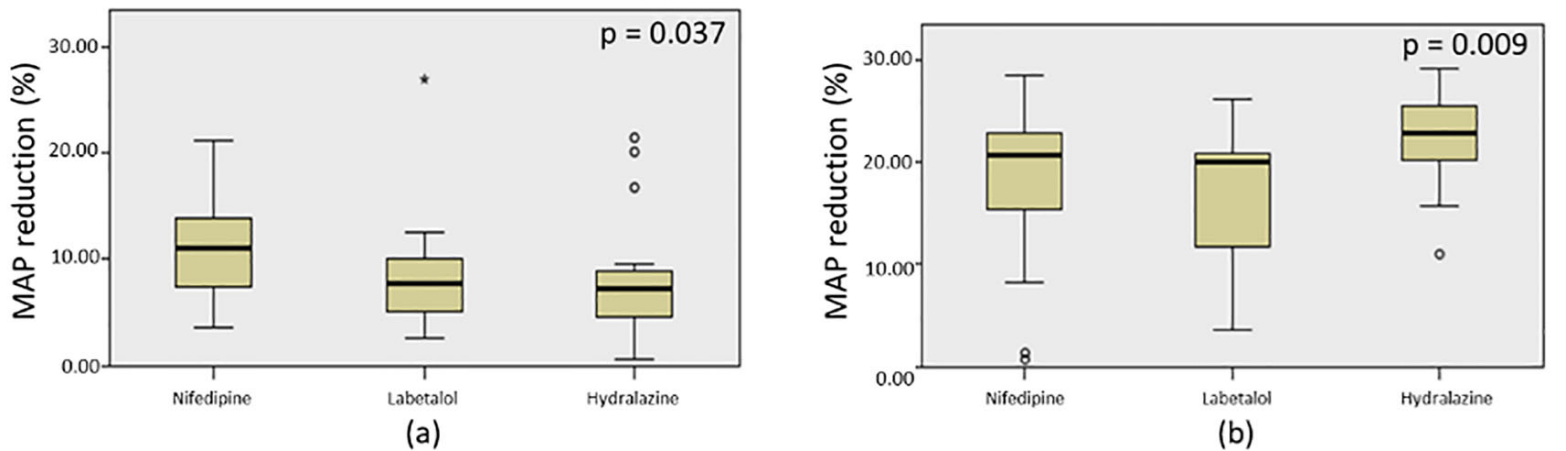
Kruskal-Wallis test on MAP reduction by nifedipine, labetalol, and hydralazine when administered in a single dose (a) and triple dose (b).

To compare the effectiveness among the three drugs, N-gain values were calculated based on the MAP reduction and presented in Table 5. The results showed that nifedipine, labetalol, and hydralazine in a single dose had N-gain values of 0.575 (58%); 0.421 (42%); and 0.408 (41%), respectively. This means, according to Melzer criteria, ^7^ these three drugs had medium effectiveness in lowering blood pressure to achieve the target MAP (20%). Meanwhile, if referred to Hake criteria, ^8^ nifedipine is highly effective, while labetalol and hydralazine are less effective in achieving a 20% MAP reduction. We also found significant difference of the MAP among the three drugs when administered in a single dose with p=0.037 based on Kruskal-Wallis test ( Figure 6). According to pairwise comparative analysis, only hydralazine and nifedipine appeared to have significant difference with p=0.048 ( Table 6). While the effectiveness of nifedipine was not significantly different with that of labetalol (p=0.148), also shown by hydralazine versus labetalol (p=1.000) ( Table 6).

**Table 5.** N-gain administration of one dose and triple doses of the test drug.

Dose	Type of anti-hypertensive	N-gain	
		Mean	Std. Error
Single dose	Nifedipine	0.574	0.062
	Labetalol	0.421	0.058
	Hydralazine	0.408	0.060
Triple dose	Nifedipine	0.900	0.034
	Labetalol	0.851	0.045
	Hydralazine	1.112	0.060

**Table 6.** Pairwise comparisons of the tested anti-hypertensives at single and triple doses administration.

Dose	Comparison	Test statistic	Std. error	Std. test statistic	adj. p-value
Single	Hydralazine vs. labetalol	2.450	5.523	0.444	1.000
	Hydralazine vs. nifedipine	13.300	5.523	2.408	0.048 *
	Labetalol vs. nifedipine	10.850	5.523	1.965	0.148
Triple	Hydralazine vs. labetalol	5.650	5.523	1.023	0.919
	Hydralazine vs. nifedipine	-16.700	5.523	-3.24	0.007 **
	Labetalol vs. nifedipine	-11.050	5.523	-2.001	0.136

* The mean difference is significant at p<0.05.

** The mean difference is significant at p<0.01.

After the administration of three doses of nifedipine, labetalol, and hydralazine within 60 minutes, the N-gain values were calculated based on the MAP reduction ( Table 5). The mean of N-gain were 0.900 (90%); 0.851 (85%); and 1.113 (111%) for nifedipine, labetalol, and hydralazine, respectively. On the basis of both Melzer and Hake criteria, ^7^^,^^8^ all the three drugs had highly effective in lowering blood pressure to achieve the target MAP of 20%. Kruskal-Wallis test revealed that the MAP reduction by all drugs were significantly different after three consecutive doses administration (p=0.009) ( Figure 6). Based on pairwise comparisons, there were no difference in the effectiveness between labetalol and nifedipine (p=0.919); and between nifedipine and hydralazine (p=0.136) ( Table 6). Significantly difference of MAP reduction was observed between labetalol and hydralazine (p=0.007) ( Table 6).

### Secondary outcome: side effects

The presence of ten side effects (tachycardia, hypotension, foetal heart rate abnormality, nausea, vomit, dizzy, palpitation, headache, chest pain, and tachypnoea) was assessed following the administration of all three drugs. No patients treated with labetalol and hydralazine complained the side effect. Among patients treated with nifedipine, no side effects were reported by the patient except tachycardia.

## Discussion

To date, ACOG has recommended three types of first-line drugs that can be used as emergency management of hypertension in pregnancy: oral nifedipine, intravenous labetalol, and intravenous hydralazine. The blood pressure targets to be achieved in patients with hypertensive emergencies in pregnancy differ from one guideline to another. ^9^ The latest ACOG bulletin guidelines did not mention the target blood pressure and only mentioned the blood pressure threshold that clinicians should to achieve. This present study was the first to report the direct comparison of the effectiveness of nifedipine, labetalol, and hydralazine together. In our study, a 20-25% decrease in MAP from baseline was considered the target blood pressure to be achieved. We also processed the data numerically so that a decrease of 1 mmHg was calculated and analysed to produce a detailed outcome. The blood pressure and pulse rate of the patients at the baseline are similar to previous studies studying the severe preeclampsia. ^9^^–^^12^ No characteristic differences were observed among nifedipine, labetalol, and hydralazine groups in the present study.

Our data suggested that oral nifedipine and intravenous labetalol and hydralazine regimens can be used to treat hypertensive emergencies in pregnancy. If 20-25% reduction of MAP is used as the target, where three doses was set as the maximum, the intravenous hydralazine monotherapy had the lowest failure rate (1 patient). In contrast, intravenous labetalol monotherapy had a failure rate 7 patients (35%, 7/20 patients). As reported previously, labetalol group had relatively low efficacy and required cross-treatment or alternative treatment. ^10^ It is worth noting that the target for MAP reduction may vary across hospitals. Hence, further processing of these ordinal data was not carried out.

Based on the data of systolic pressure following post-administration time, nifedipine had maximum effectiveness by one dose of administration, but experienced a rebound after 4 hours. As in the labetalol group, the blood pressure reduction peaked twice; at post-20 and -60 minutes administration. Patients in hydralazine group had the lowest blood pressures at three points of measurement: at post-20, -40, and -60 minutes administration. These findings suggest that titration dose is required. Comparative analysis among the three drugs using a single dose yielded a statistical significance of p=0.037. When administered with a single dose, nifedipine performed the best, followed by labetalol and hydralazine and no significant difference of efficacy between labetalol and nifedipine. The result is similar to the finding from a previous study ^10^ where labetalol and nifedipine had no significant different in reduction of the blood pressure. Rapid reduction of the blood pressure in nifedipine group in our study also similar with previous study ^13^ where the reduction of the blood pressure was achieved faster by nifedipine compared with hydralazine. ^13^ Both nifedipine and hydralazine work by resulting vasodilating effects. ^14^^,^^15^ However, hydralazine has more effect on the arteries than veins ^15^ while nifedipine, as a calcium channel blocker, causes overall vasodilation, thereby leading to a decrease in peripheral vascular resistance. ^14^ Taken altogether, nifedipine could be the most efficacious when administered at a single dose.

According to the ACOG guidelines, the maximum dose of emergency antihypertension is 3 times administration within 60 minutes. The comparative analysis revealed that hydralazine yielded the most optimum reduction of blood pressure, followed by nifedipine and labetalol, respectively. This could be observed through the significant reduction of blood pressure on the first until the fourth measurements.

We did not witness any adverse effects experienced by the mothers or infants following the drug administration, in which our data are similar to that of reported. ^16^^–^^18^ Nonetheless, other studies reported the occurrence of nausea and vomiting as adverse effects of labetalol (16%) or nifedipine (8%). ^10^ In another study, patients in labetalol and nifedipine group experienced dizziness and/or headaches (24% vs 12%), palpitations (8% vs 4%), and foetal heart rate abnormalities (4% vs 8%). ^9^ These different findings on side effect might be due to the maximum dose used in both foregoing studies, which was 5 doses. ^9^^,^^10^

The strengths of this study include the multicentred randomized controlled study design, intensive monitoring, and repeated measurements up to seven times. However, this study is limited by the fact that some of the hospitals only used single blinding because labetalol and hydralazine are new to some doctors. We also did not consider the circadian rhythms in our study and this might affect the blood pressure. Some of the side effects were measured subjectively such as nausea, vomit, dizzy, palpitation, headache and chest pain. However, the methods were exactly the same in all groups.

## Conclusions

Our data suggested that nifedipine is the most effective in reducing blood pressure using a single dose administration during hypertensive emergency in severe preeclampsia. The lowering blood pressure effect of nifedipine was not significantly different to labetalol but was significantly superior as compared with hydralazine. Nifedipine had a significant rebound phenomenon in blood pressure after four hours of drug administration, hence the necessity of re-administering antihypertensives. In maintaining the low blood pressure, hydralazine was significantly more effective than labetalol and no different effectiveness between hydralazine and nifedipine. All drugs are considered safe after triple dose administration within one hour. 

Based on the present study, in high resource settings, hydralazine is recommended to treat severe preeclampsia cases with hypertensive emergency since it reduces the blood pressure rapidly and has no rebound phenomenon. We recommend to use nifedipine in the low resource settings since it is cheap and easy to use and store while still has good ability to reduce the blood pressure. However, blood pressure maintenance is required because it might rebound after approximately six hours.

## Data Availability

Figshare: ‘Effectiveness of nifedipine, labetalol, and hydralazine as emergency antihypertension in severe preeclampsia’. DOI: https://doi.org/10.6084/m9.figshare.20933785. ^19^ This project contains the following underlying data:
- Master Data.xlsx [Table containing the raw data of the study]. Figshare: CONSORT checklist for Effectiveness of nifedipine, labetalol, and hydralazine as emergency antihypertension in severe preeclampsia. DOI: https://doi.org/10.6084/m9.figshare.21443028. ^20^ Data are available under the terms of the Creative Commons Attribution 4.0 International license (CC-BY 4.0).
