# Supplementary material for: Effectiveness of nifedipine, labetalol, and hydralazine as emergency antihypertension in severe preeclampsia: a randomized control trial
Source: F1000Res. 2023 Apr 27;11:1287. Originally published 2022 Nov 10. [Version 2] doi: 10.12688/f1000research.125944.2 (PMC10238817; doi:10.12688/f1000research.125944.2)
Supplement: Peer review history

# Peer review: Reviewer response for version 2

**DOI:** 10.5256/f1000research.144510.r171164

**Published:** 2023-06-02

**Authors:** Jon Wantania, Windy Wariki

**Affiliations:** 1 Department of Obstetric and Gynaecology, Maternal Foetal Medicine Division, Medical Faculty, Sam Ratulangi University, Manado, North Sulawesi, Indonesia; 2 Sam Ratulangi University, Manado, North Sulawesi, Indonesia

Thank you for revision of your manuscript.

Is the work clearly and accurately presented and does it cite the current literature?

Partly

If applicable, is the statistical analysis and its interpretation appropriate?

I cannot comment. A qualified statistician is required.

Are all the source data underlying the results available to ensure full reproducibility?

Yes

Is the study design appropriate and is the work technically sound?

Partly

Are the conclusions drawn adequately supported by the results?

Partly

Are sufficient details of methods and analysis provided to allow replication by others?

Partly

Reviewer Expertise:

John Wantania : Maternal Fetal Medicine ;                 Windy Mariane Virenia Wariki :  Clinical Epidemiologist

We confirm that we have read this submission and believe that we have an appropriate level of expertise to confirm that it is of an acceptable scientific standard.

# Peer review: Reviewer response for version 2

**DOI:** 10.5256/f1000research.144510.r171163

**Published:** 2023-05-02

**Authors:** Talha Bin Emran

**Affiliations:** 1 Department of Pharmacy, BGC Trust University Bangladesh, Chittagong, Bangladesh

Authors addressed all of my comments. The revised manuscript can be accepted for indexing.

Is the work clearly and accurately presented and does it cite the current literature?

Yes

If applicable, is the statistical analysis and its interpretation appropriate?

Yes

Are all the source data underlying the results available to ensure full reproducibility?

Yes

Is the study design appropriate and is the work technically sound?

Yes

Are the conclusions drawn adequately supported by the results?

Yes

Are sufficient details of methods and analysis provided to allow replication by others?

Yes

Reviewer Expertise:

Immunology, Alternative Medicine, Public Health, Bioinformatics

I confirm that I have read this submission and believe that I have an appropriate level of expertise to confirm that it is of an acceptable scientific standard.

# Peer review: Reviewer response for version 1

**DOI:** 10.5256/f1000research.138303.r155526

**Published:** 2022-12-02

**Authors:** Jon Wantania, Windy Wariki

**Affiliations:** 1 Department of Obstetric and Gynaecology, Maternal Foetal Medicine Division, Medical Faculty, Sam Ratulangi University, Manado, North Sulawesi, Indonesia; 2 Sam Ratulangi University, Manado, North Sulawesi, Indonesia

Thank you for your hard work. As a reviewer, I appreciate the efforts of the authors. The methods and research results presented are excellent and it hoped that great benefits obtained from this article. However, there are still some minor issues to address, which are listed below, in general:

- This article is useful, but still there are several weaknesses that need to be considered.
- The introduction section should describe in more depth what is the novelty and urgency of this study.
- The methods, and interpretations of the statistical analysis are not carefully presented. Therefore, further consultations with biostatistician or epidemiologist may potentially improve this article.
- The authors may not be careful enough in writing due to some mistakes in grammar and inconsistency in writing.

**Introduction**

 Paragraph 4: The authors mentioned about the recommendations for antihypertension drugs in preeclampsia and stated, “Multiple drugs are included and excluded from the list of drugs of choice for the treatment of severe preeclampsia”. This sentence is a little unclear. Could you please clarify exactly what you mean about multiple drugs being included and also excluded as well? The authors should modify the sentence so that it is easier for the reader to understand.

 Paragraph 4: In the third sentence the authors explained the dosage form of hydralazine, without writing down the dosage form of labetalol.

 Paragraph 5: The authors stated: “Hydralazine and labetalol are considered to have a balanced effectiveness and side effect in lowering blood pressure in preeclampsia”, and “Another study comparing nifedipine and labetalol…” Here, the authors should explain if the indicator used to measure “lowering blood pressure” were the same, for example the mean arterial pressure (MAP), and need to confirm quantitatively how much the blood pressure declined so that the reader can judge that they are comparable.

 Paragraph 6: In the objective of this study, the authors mentioned the dosage form of nifedipine and labetalol. The authors should consistently explain the dosage form of hydralazine as well, bearing in mind that nifedipine and labetalol have different dosage forms.

**Methods**

 Study setting and registration

 In line with the subtitle above including registration, the authors should explain matters related to registration in this section instead of explaining it in the ethical considerations section. However, if the authors should explain this in ethical considerations section, can “registration” in this subtitle be eliminated?

 Patient recruitment and criteria

 In the second sentence the authors stated “The minimal sample size of the study was 13 patients”. This is different from the explanation in the Results section that the total number of samples was 60. Does it mean that the results of the minimum sample size calculation are 13 patients in each group?

 The minimum sample calculation formula used by the authors is for two groups. Considering that this research was an unpaired numerical comparative study conducted on three groups with measurements of seven times, it would be more appropriate if the authors calculated the number of samples based on the above considerations.

 Blinding and study procedure

 The authors are suggested to explain in more detail about the following sentence “For example, the patient who arrived first and second to the hospital should be given kit no 41 and 23”. Furthermore, the authors also need to describe who are the “health workers” specifically, mentioned in this sentence “Health workers were instructed to administrate one pair of drug….” with reference to Peraturan Menteri Kesehatan Republik Indonesia Nomor 13 Tahun 2020.

 Endpoints:

 The authors stated “The blood pressure was measured seven times...” As a suggestion, this sentence should be more suitable when explained in the subtitle “Blinding and study procedure” above.

 Statistical analysis

 The authors have explained that a normality test has been carried out, therefore it is advised to write down the name of the normality test used, for example by taking into account the number of samples, or other considerations or assumptions.

**Results**

 Figure 1: In the last column of the “Analysis” section, it was written that the total number of respondents was 49 patients, quite different from the explanation in the Results section that 60 patients were analyzed. It is suggested that the authors need to be clearer, for example, when and for what to analyzing 60 patients, or only 49 patients.

 Required time for nifedipine, labetalol, and hydralazine group achieved the MAP

 The authors have explained that the results regarding the time required for the three drugs to reach the target MAP are presented in Figure 1. However, the results shown in figure 1 are different from this explanation.

 Nifedipine (page 6)

 In this section, the author has directed the reader for three times to see the results in Table 2, even though all the data in this section are only showed in table 2. As a suggestion, it would be better if Table 2 was written only once, for example at the beginning or end of a paragraph of the explanation.

 Sentence stating “Nifedipine decrease the MAP 15.61 mmHg, 5.46 mmHg and 12.00 mmHg 20 min post the first…”. However, 5.46 mmHg and 12.00 mmHg are not found in Table 2. Similar with the sentence sixth which is in Table 2 the p=0.010 and p=0.024 are not found.

 Table 2

 On the footnote of table 2 was written the mean difference is significant at p<0.005. But in this table, there is no p-value less than 0.05 (p<0.05) which means that this mark (*) no need to be mentioned in the footnote.

 Page 7: The description below Figure 2 where the author wants to explain the changes of mean systolic, diastolic and MAP. The authors are asked to be more careful in presenting the results where the appearance of Figure 2 inconsistent from this explanation. Figure 2 is a comparison of the three types of drugs in achieving MAP.

 Figure 2:

 It would be more informative and flowing if the appearance of Figure 2 and its description are in the “Effectiveness comparisons of nifedipine, labetalol, and hydralazine” section compared to the current view which is in the section or explanation of nifedipine.

***Labetalol***

 The authors are advised to revise the figure number that is not in accordance with the explanation about labetalol, which is different from what is presented in Figure 3.

***Hydralazine***

 In Table 4, the results of the analysis to explain the decrease in systolic blood pressure were not found. Also, Figure 4 is a description of labetalol, not hydralazine.

 Page 8: Explanation of the results of Kruskal-Wallis test is not present in the Figure 5, but in Figure 6.

 Table 7: Suggestion for the authors regarding reported side effects where there was only one namely tachycardia, it would be better if the result is presented in a narrative manner.

**Discussion**

 Discussion section is still having major problems, addition information requested on strength and limitation are not clearly explain.

 The authors are lacking in explanation the agreements and disagreements with findings from other studies.

 Limitations

 The limitation in methodological should be clearly explained and address as one of the limitations of this study.

 The authors are suggested to give specific recommendations to the reader as respond to key findings.

Is the work clearly and accurately presented and does it cite the current literature?

Partly

If applicable, is the statistical analysis and its interpretation appropriate?

I cannot comment. A qualified statistician is required.

Are all the source data underlying the results available to ensure full reproducibility?

Yes

Is the study design appropriate and is the work technically sound?

Partly

Are the conclusions drawn adequately supported by the results?

Partly

Are sufficient details of methods and analysis provided to allow replication by others?

Partly

Reviewer Expertise:

John Wantania : Maternal Fetal Medicine ;                 Windy Wariki :  Clinical Epidemiologist

We confirm that we have read this submission and believe that we have an appropriate level of expertise to confirm that it is of an acceptable scientific standard, however we have significant reservations, as outlined above.

# Response

**Published:** 2023-02-16

**Authors:** Donel S

**Affiliations:** Universitas Riau, Indonesia

Thank you for your hard work. As a reviewer, I appreciate the efforts of the authors. The methods and research results presented are excellent and it hoped that great benefits obtained from this article. However, there are still some minor issues to address, which are listed below, in general:

 This article is useful, but still there are several weaknesses that need to be considered.

 The introduction section should describe in more depth what is the novelty and urgency of this study.

 The methods, and interpretations of the statistical analysis are not carefully presented. Therefore, further consultations with biostatistician or epidemiologist may potentially improve this article.

 The authors may not be careful enough in writing due to some mistakes in grammar and  inconsistency in writing.

 RESPONSES:  Thank you for reviewing our manuscript. The comments and suggestions have improved the quality of our current manuscript significantly.

 We have added the required information in the methods as requested. Also, we have corrected some typos in the numbering related to the statistics. We have corrected the labels of the figures. The connection between the figures and the explanation texts have been corrected and checked for the correctness.

**Introduction**

 Paragraph 4: The authors mentioned about the recommendations for antihypertension drugs in preeclampsia and stated, “Multiple drugs are included and excluded from the list of drugs of choice for the treatment of severe preeclampsia”. This sentence is a little unclear. Could you please clarify exactly what you mean about multiple drugs being included and also excluded as well? The authors should modify the sentence so that it is easier for the reader to understand.

 RESPONSES:  We have revised the sentence.

 Paragraph 4: In the third sentence the authors explained the dosage form of hydralazine, without writing down the dosage form of labetalol.

 RESPONSES:  Have been included: “ **Intravenous** labetalol and intravenous hydralazine have long been considered as first-line therapy in hypertensive emergencies, severe hypertension in pregnant women, and in the postpartum period”.

 Paragraph 5: The authors stated: “Hydralazine and labetalol are considered to have a balanced effectiveness and side effect in lowering blood pressure in preeclampsia”, and “Another study comparing nifedipine and labetalol…” Here, the authors should explain if the indicator used to measure “lowering blood pressure” were the same, for example the mean arterial pressure (MAP), and need to confirm quantitatively how much the blood pressure declined so that the reader can judge that they are comparable.

 RESPONSES:  We have added more detailed of the information related to the studies.

 Paragraph 6: In the objective of this study, the authors mentioned the dosage form of nifedipine and labetalol. The authors should consistently explain the dosage form of hydralazine as well, bearing in mind that nifedipine and labetalol have different dosage forms.

 RESPONSES:  Have been included: “Therefore, this study was conducted to compare the effectiveness of oral nifedipine, and intravenous labetalol and **intravenous** hydralazine as antihypertensives in severe preeclampsia.”

**Methods**

 Study setting and registration

 In line with the subtitle above including registration, the authors should explain matters related to registration in this section instead of explaining it in the ethical considerations section. However, if the authors should explain this in ethical considerations section, can “registration” in this subtitle be eliminated?

 RESPONSES:  Thank you for the suggestion. We have changed the subheading to: “Study setting” only and moved the “registration” into: “Ethical considerations and registration”

 Patient recruitment and criteria

 In the second sentence the authors stated “The minimal sample size of the study was 13 patients”. This is different from the explanation in the Results section that the total number of samples was 60. Does it mean that the results of the minimum sample size calculation are 13 patients in each group? RESPONSES: 13 patients are minimal sample size for each group. Therefore, we have revised to: “The minimal sample size of the study was 13 patients for each arm.”

 The minimum sample calculation formula used by the authors is for two groups. Considering that this research was an unpaired numerical comparative study conducted on three groups with measurements of seven times, it would be more appropriate if the authors calculated the number of samples based on the above considerations.

 RESPONSES: We have checked again and we confirm that this formula also can be used for three groups.

 Blinding and study procedure

 The authors are suggested to explain in more detail about the following sentence “For example, the patient who arrived first and second to the hospital should be given kit no 41 and 23”.  

 RESPONSES: We have revised the sentence and we believe it is clear now. “For example, if the list from the software run was 41, 23, 4 and soon, the patient who arrived first, second and third to the hospital should be given kit no 41, 23 and 4, respectively.”

 Furthermore, the authors also need to describe who are the “health workers” specifically, mentioned in this sentence “Health workers were instructed to administrate one pair of drug….” with reference to Peraturan Menteri Kesehatan Republik Indonesia Nomor 13 Tahun 2020.

 RESPONSES: They were doctors. And the text has been revised: “The enrolment of the patient was conducted by the doctors in each centre. The doctors have been informed about the study and have been trained. The doctors and patients did not know the drugs contained in the kit.”

 Endpoints:

 The authors stated “The blood pressure was measured seven times...” As a suggestion, this sentence should be more suitable when explained in the subtitle “Blinding and study procedure” above.

 RESPONSES:  Thank you for the suggestion. The sentence has been moved within “Blinding and study procedure” subheading.

 Statistical analysis

 The authors have explained that a normality test has been carried out, therefore it is advised to write down the name of the normality test used, for example by taking into account the number of samples, or other considerations or assumptions.

 RESPONSES: We have added the information of the test used to measure the normality of the data. We added: “The normality of data was measured using the Kolmogorov-Smirnov test.”

**Results**

 Figure 1: In the last column of the “Analysis” section, it was written that the total number of respondents was 49 patients, quite different from the explanation in the Results section that 60 patients were analyzed. It is suggested that the authors need to be clearer, for example, when and for what to analyzing 60 patients, or only 49 patients.

 RESPONSES: Thank you for questioning this. We have added the information related to this. We add: “Out of 60 patients, 11 patients did not achieve the MAP target after three doses of the tested drugs and therefore were excluded for effectiveness analysis of nifedipine, labetalol, and hydralazine to reduce the blood pressure.”

 Required time for nifedipine, labetalol, and hydralazine group achieved the MAP

 The authors have explained that the results regarding the time required for the three drugs to reach the target MAP are presented in Figure 1. However, the results shown in figure 1 are different from this explanation.

 RESPONSES: Thank you pointing this mistake. It should be Figure 2. Also it has been changed to “Required **dose** for nifedipine, labetalol, and hydralazine to achieve MAP target” “Data regarding the dose required for nifedipine, labetalol, and hydralazine to reach the target MAP (20% reduction) are presented in Figure 2.”

 Nifedipine (page 6)

 In this section, the author has directed the reader for three times to see the results in Table 2, even though all the data in this section are only showed in table 2. As a suggestion, it would be better if Table 2 was written only once, for example at the beginning or end of a paragraph of the explanation.

 RESPONSES: Now it only mentioned only once in the beginning.

 Sentence stating “Nifedipine decrease the MAP 15.61 mmHg, 5.46 mmHg and 12.00 mmHg 20 min post the first…”. However, 5.46 mmHg and 12.00 mmHg are not found in Table 2. Similar with the sentence sixth which is in Table 2 the p=0.010 and p=0.024 are not found.

 RESPONSES:  We appreciated with this correction. We have corrected the sentences: “Nifedipine decreased the MAP 15.61 mmHg, 6.37 mmHg and 2.90 mmHg 20 min post the first, second and the third dose of nifedipine. The MAP reduction on 20 min post the first dose was significant statistically with p<0.001.”

 Table 2

 On the footnote of table 2 was written the mean difference is significant at p<0.005. But in this table, there is no p-value less than 0.05 (p<0.05) which means that this mark (*) no need to be mentioned in the footnote.

 RESPONSES:  Now the * has been deleted. And ** has been changed to “*The mean difference is significant at p<0.01.” The * in the table also have been deleted. Thank you.

 Page 7: The description below Figure 2 where the author wants to explain the changes of mean systolic, diastolic and MAP. The authors are asked to be more careful in presenting the results where the appearance of Figure 2 inconsistent from this explanation. Figure 2 is a comparison of the three types of drugs in achieving MAP.

 RESPONSES: Thank you for catch this mistake. We realized that there was a shift of the labels of the figures. This should be Figure 3 not Figure 2. Now it has been corrected into Figure 3. The revised texts: “The changes of the mean systolic, diastolic and MAP are presented in **Figure 3**. The data suggested there was a sharp decrease of systolic and diastolic after the first dose of nifedipine and the blood pressure kept decrease until 20 min after the third dose (after 2 hours of first dose) but it started to bounce again afterward ( **Figure 3**).”

 Figure 2:

 It would be more informative and flowing if the appearance of Figure 2 and its description are in the “Effectiveness comparisons of nifedipine, labetalol, and hydralazine” section compared to the current view which is in the section or explanation of nifedipine.

 RESPONSES: We agree with this suggestion because it is hard to follow the texts and the Figure 2.

 We have re-requested the layout editor to move Figure 2 directly under the subheading: “Required dose for nifedipine, labetalol, and hydralazine to achieve MAP target”.

***Labetalol***

 The authors are advised to revise the figure number that is not in accordance with the explanation about labetalol, which is different from what is presented in Figure 3.

 RESPONSES: As we responded previously, we realized that there was a shift of the labels of the figures. Now all have been corrected.

***Hydralazine***

 In Table 4, the results of the analysis to explain the decrease in systolic blood pressure were not found. Also, Figure 4 is a description of labetalol, not hydralazine.

  RESPONSES: The systolic data was not distributed normally and therefore the Friedmann test and this analysis produced one p-value only and no 95%CI. To ensure this is clear, we have included the information as a note under the Table 4. “ ^c^ Analysed using Friedmann test.”

 As we stated previously, all labels of the figures have been rechecked and corrected.

 Page 8: Explanation of the results of Kruskal-Wallis test is not present in the Figure 5, but in Figure 6.

 RESPONSES: All labels of the figures have been rechecked and corrected.

 Table 7: Suggestion for the authors regarding reported side effects where there was only one namely tachycardia, it would be better if the result is presented in a narrative manner.

 RESPONSES: Now Table 7 has been deleted as suggested and we have added “side effects” after the secondary outcome.

**Discussion**

 Discussion section is still having major problems, addition information requested on strength and limitation are not clearly explain.

 RESPONSES: We have provided the information related to the strengths and limitations of the study.

 The authors are lacking in explanation the agreements and disagreements with findings from other studies.

 RESPONSES: Thank you for your suggestions. Unfortunately, available studies are limited and therefore it is not possible to compare with previous studies.

 Limitations

 The limitation in methodological should be clearly explained and address as one of the limitations of this study.

 The authors are suggested to give specific recommendations to the reader as respond to key findings.

 RESPONSES:  We have listed all the limitations of the study. We also have added specific recommendation from the results of the study. We added: “ Based on the present study, in high resource settings, hydralazine is recommended to treat severe preeclampsia cases with hypertensive emergency since it reduces the blood pressure rapidly and has no rebound phenomenon. We recommend to use nifedipine in the low resource settings since it is cheap and easy to use and store while still has good ability to reduce the blood pressure. However, blood pressure maintenance is required because it might rebound after approximately six hours.”

# Peer review: Reviewer response for version 1

**DOI:** 10.5256/f1000research.138303.r155523

**Published:** 2022-11-14

**Authors:** Talha Bin Emran

**Affiliations:** 1 Department of Pharmacy, BGC Trust University Bangladesh, Chittagong, Bangladesh

The study assessed the effectiveness of nifedipine, labetalol, and hydralazine as emergency antihypertension in severe preeclampsia (SP). SP is a significantly important health problem in both developed and developing countries. My comments:

- The study was well-designed and conducted with balanced conclusions.
- The title of the study is clear and balanced however the information on RCT is needed such as: “Effectiveness of nifedipine, labetalol, and hydralazine as emergency antihypertension in severe preeclampsia: a randomized control trial”.
- The structured abstract contains all important information, including the registry number.
- The introduction section provides the importance and significance of the problem as well as the scientific gap. The aim of the study is also clearly stated. 
- The study setting is clearly stated although the justification for choosing those hospitals might be helpful.
- The sample size is relatively small, but the justification and calculation are provided on how the sample was calculated.
- Double blinding and randomization were conducted to avoid bias.
- The procedures are explained adequately but the information on whether the healthcare workers received the training from the researchers is important to ensure that they worked following the guideline.
- The Results assisted with a flowchart, and this helps the readers to understand each step of enrolment, allocation, follow-up, and analysis.
- The baseline of the data of the patients between the group are relatively homogeneous.
- The comparations between drugs were conducted for each endpoint and should be appreciated.
- Table 7. The information in this table can basically be written in two sentences only since there were no side effects in the labetalol and hydralazine groups. Also “Secondary outcome” could be changed to “Side effect.”
- Discussion and conclusion are balanced; the study's strengths and limitations are discussed. However, some suggestions to measure soluble fms-like tyrosine kinase 1, endothelin-1, and soluble endoglin likely do not support the data of this study.

Is the work clearly and accurately presented and does it cite the current literature?

Yes

If applicable, is the statistical analysis and its interpretation appropriate?

Yes

Are all the source data underlying the results available to ensure full reproducibility?

Yes

Is the study design appropriate and is the work technically sound?

Yes

Are the conclusions drawn adequately supported by the results?

Yes

Are sufficient details of methods and analysis provided to allow replication by others?

Yes

Reviewer Expertise:

Immunology, Alternative Medicine, Public Health, Bioinformatics

I confirm that I have read this submission and believe that I have an appropriate level of expertise to confirm that it is of an acceptable scientific standard.

# Response

**Published:** 2023-02-16

**Authors:** Donel S

**Affiliations:** Universitas Riau, Indonesia

The study assessed the effectiveness of nifedipine, labetalol, and hydralazine as emergency antihypertension in severe preeclampsia (SP). SP is a significantly important health problem in both developed and developing countries. My comments: The study was well-designed and conducted with balanced conclusions.

 RESPONSES: Thank you for your appreciation.

 The title of the study is clear and balanced however the information on RCT is needed such as: “Effectiveness of nifedipine, labetalol, and hydralazine as emergency antihypertension in severe preeclampsia: a randomized control trial”.

 RESPONSES: We have revised the tittle by adding: “: a randomized control trial”

 The structured abstract contains all important information, including the registry number.

 The introduction section provides the importance and significance of the problem as well as the scientific gap. The aim of the study is also clearly stated. 

 RESPONSES: Thank you for your appreciation.

 The study setting is clearly stated although the justification for choosing those hospitals might be helpful.

 RESPONSES: The reasons have been provided: “These hospitals were chosen because they are affiliated with Faculty of Medicine, Universitas Riau.”

 The sample size is relatively small, but the justification and calculation are provided on how the sample was calculated.

 Double blinding and randomization were conducted to avoid bias.

 RESPONSES: Thank you for your appreciation.

 The procedures are explained adequately but the information on whether the healthcare workers received the training from the researchers is important to ensure that they worked following the guideline.

 RESPONSES: We have added the information that the healthcare workers have been trained: “The doctors have been informed about the study and have been trained.” 

 The Results assisted with a flowchart, and this helps the readers to understand each step of enrolment, allocation, follow-up, and analysis.

 The baseline of the data of the patients between the group are relatively homogeneous.

 The comparations between drugs were conducted for each endpoint and should be appreciated

 RESPONSES: Thank you for your appreciation.

 Table 7. The information in this table can basically be written in two sentences only since there were no side effects in the labetalol and hydralazine groups. Also “Secondary outcome” could be changed to “Side effect.”

 RESPONSES: Thank you for the suggestion. Now Table 7 has been deleted and we have added “side effects” after the secondary outcome.

 Discussion and conclusion are balanced; the study's strengths and limitations are discussed. However, some suggestions to measure soluble fms-like tyrosine kinase 1, endothelin-1, and soluble endoglin likely do not support the data of this study.

 RESPONSE:  Thank you for the suggestion. This sentence has been deleted.
